# Hidradenitis suppurativa: Coexistence or dermatological extraintestinal manifestation of Crohn's disease?

**DOI:** 10.3389/fmed.2023.1151370

**Published:** 2023-03-23

**Authors:** Rafael Luís Luporini, Pâmela Cristina Bellaz Do Amaral Campos Silva, Miguel Regueiro

**Affiliations:** ^1^Department of Medicine, Federal University of São Carlos (UFSCar), São Carlos, Brazil; ^2^General Surgery, Santa Casa de São Carlos, São Carlos, Brazil; ^3^University Hospital of the Federal University of São Carlos, São Carlos, Brazil; ^4^Department of Medicine, Cleveland Clinic Lerner College of Medicine, Case Western Reserve University, Cleveland, OH, United States

**Keywords:** hidradenitis suppurativa, Crohn's disease, dermatological extraintestinal manifestation, perianal Crohn's disease, inflammatory bowel diseases, chronic inflammatory diseases

## Introduction

Crohn's disease (CD) is an inflammatory bowel disease (IBD) of the gastrointestinal tract (GIT) with a complex, not yet fully understood pathogenesis that includes genetic aspects, environmental influences (including the gut microbiota), and dysregulation of innate and adaptive immune responses (1). Although GIT is the main source of CD symptoms, there may also be extraintestinal manifestations (EIM), including dermatologic conditions such as pyoderma gangrenosum and erythema nodosum (2).

Hidradenitis suppurativa (HS) or acne inversa is also a chronic, recurrent and debilitating inflammatory and immune-mediated disease of the pilosebaceous unit. Painful subcutaneous nodules, pustules, abscesses, fistulas, and scar tissue are the most common manifestations. HS preferentially affects skin folds, especially axillary, inguinal, perianal, perineal, gluteal, inframammary, and anogenital conditions (2–6). The prevalence of HS is 0.1 to 1%, with onset at puberty and adolescence, more frequent in women (7, 8). The pathogenesis of HS is multifactorial, being an autoinflammatory process triggered by a perifollicular lymphocytic infiltrate leading to occlusion of the excretory ducts of the apocrine glands, dilatation and rupture of the pilosebaceous unit. The result is increased inflammation with the formation of tunnels connected to the skin surface and filled with debris. The local microbiota may play a role in the dysregulation of the innate immune system (4–6). Genetic factors also play a role, with a familial disorder present in 34% of cases (9). Several known autoinflammatory syndromes are associated with HS including PASH (pyoderma gangrenosum, acne, and HS), a potentially debilitating disorder (10). Although unclear, studies have demonstrated the association of HS with dysregulation of the interleukin (IL) 12/23 and tumor necrosis factor-α (TNF-α) inflammatory pathways (8).

Several studies have shown cases of associations between HS and CD, but a direct relationship was not clear (9, 11, 12). Recent studies have found clinical, pathogenic, and immunogenic features common to CD and HS that may support the theory that HS is an extraintestinal manifestation (EIM) of CD. The questions that remain are: Is HS actually metastatic CD? Are HS and Crohn's disease similar immune-mediated diseases? Is HS an EIM of CD?

## Studies associating CD and HS

Case reports registered the occurrence of CD and HS in the same patient, including Shalom et al. (13) who detected 26 cases of CD associated with HS among 3,207 patients diagnosed with HS and showed a significant association (*p* = 0.01) between the two diseases, interestingly, there was no association with ulcerative colitis. Janse et al. (14) discovered a prevalence of HS in 6.8–10.6% of patients with IBD vs. 1–2% in the general population. Moreover, other studies have shown that patients with CD have a significantly increased risk of HS compared with the general population. In a population-based cohort study from Minnesota evaluating 679 patients with IBD, 1.2% with HS had a 9-fold higher relative risk than the general population, with CD (relative risk 12-fold) predominating relative to ulcerative colitis (UC) (relative risk seven-fold) (5, 8). Kamal et al. (2) demonstrated a much more common association of HS with CD than UC. There are some studies that indicate an even higher prevalence of HS in patients with IBD—between 16 and 23%. It is important to consider reporting bias as these reports are from IBD centers and patient's self-report HS diagnosis (8, 9, 12). Garg et al. (3) identified 51,340 patients with HS, with a prevalence of CD among these patients of 2.0% compared with 0.6% among those without HS (*p* < 0.001). HS can occur before or after the development of CD, with variable time intervals (7). Yadav et al. (8) showed that CD occurs on average 8.4 years before HS.

HS is currently considered a disease whose mechanisms are unclear in the context of IBD (15, 16). Both HS and CD include abscesses in the perineal and inguinal region, and sinus tracts, association with arthritis, and a positive response to anti-TNF-α. It also shares a common pathogenic feature with an aberrant immune response (11). In addition, smoking is a risk factor for both diseases (7).

Because of their similarity, distinguishing between perianal HS and perianal CD is a diagnostic challenge. Clinically, both may present with abscesses, sinus tracts with perianal pain, redness, itching, bleeding, and purulent secretions. Histologic examination is not always sufficient to distinguish the two, as both may show formation of granulomas and lymphoid follicles. Imaging studies, such as nuclear magnetic resonance imaging (NMR), may be helpful in the differential diagnosis (5, 11).

A NMR imaging study at HS showed some changes with predominant features that may help distinguish between the two diseases, such as “absence of characteristics” in the perianal area, absence of rectal thickening, and bilaterality of findings, in addition to subcutaneous edema, lower involvement of the anal sphincter, and sinus tract without connection to the anal canal (5, 17).

To highlight some differences between the diseases, HS fistulas do not extend to the dentate line. In CD, gastrointestinal symptoms, abdominal pain, diarrhea, iron deficiency anemia, and weight loss usually occur (5).

The immunobiologics infliximab and adalimumab have been shown to be effective in the treatment of both HS and CD (5, 18, 19). Adalimumab should be started at a stage when lesions are reversible, such as inflammatory nodules and abscesses, before lesions become irreversible, such as fistulas, sinus tracts, and scarring sequelae. This fact was confirmed in the work of Marzano et al. (20), which showed an inverse relationship between therapeutic delay and clinical response to medication in HS. Recent studies have also demonstrated the efficacy of ustekinumab in cases of HS (21, 22). It should be remembered that there are treatments for HS that should be based on the severity of the disease, and other medications such as clindamycin, rifampicin, hormonal therapies, and association with various surgical approaches can be used, from incision and drainage to control infectious foci to extensive resections to remove irreversible lesions. Important risk factors such as smoking, obesity, and improved skin hygiene should also be controlled (23).

Studies have shown that HS and CD share the same type of deregulation of immunity mechanisms, with significant increase in some IL, the main ones being IL-1, IL-6, IL-17, IL-23, and TNF-α (5, 24, 25). Giudici et al. (7) compared inflammatory changes in HS lesions and CD fistula in three patients, evidencing some similarities, such as accumulation of CD161+ T lymphocytes (associated with Th17, Th17/1, and non-classical Th1 phenotypes), and may establish a possible association between both diseases. Janse et al. (14) performing genetic evaluation, detected 2 genes *(SULT1B1* and *SULT1E1*) associated with risk of combined HS and IBD and 1 gene (*ELOVL7*) as a protective gene. The similarities in relation to clinical, histological, immune dysregulation, genetic and treatment response characteristics make the association between CD and HS very acceptable (5). Marzano et al. (26) conducted a whole-exome sequencing study of syndromic HS patients with coexisting gut and/or joint inflammation, detecting 3 genetic variants in *NOD2* gene in patients with PASH and gut inflammation. It is well-known that *NOD2* gene variants are classically associated with CD (27).

## Extraintestinal manifestations of inflammatory bowel diseases

EIM is defined as an inflammatory pathology in a patient with IBD located outside the intestine and whose pathogenesis depends on the extent/translocation of immune responses of the intestine, or is an independent inflammatory event perpetuated by IBD or that shares with it a common environmental factor or genetic predisposition (16).

CD presents several types of concomitant diseases as extraintestinal manifestations of IBD. EIM may occur in 31% of CD cases, with women predominating (1). Such manifestations may have an independent course or be associated with CD activity. These extraintestinal manifestations may involve joints (spondyloarthropathies, arthritis), eyes (uveitis, episcleritis), hepatobiliary pathways (primary sclerosing cholangitis), and skin. Dermatological manifestations include pyoderma gangrenosum, erythema nodosum, psoriasis, metastatic Crohn's disease, and Sweet syndrome (1, 28).

Orocutaneous EIM occurs in 16% of cases and can be divided into 4 distinguishing groups: (1) skin lesions with the same features as intestinal IBD (metastatic CD), (2) reactive manifestations possibly triggered by antigenic epitopes shared by intestinal IBD (erythema nodosum and pyoderma gangrenous), (3) dermatoses associated with IBD (psoriasis), and (4) secondary manifestations resulting from treatment of IBD (1). For dermatological EIMs, treatment with immunobiological therapies has been indicated (29). The fact that they improve with anti-TNF-α immunobiologicals implies a TNF-alpha-dependent mechanism for such pathologies (16).

## Discussion

Summarizing the recent findings about HS and CD, there appears to be an association between the two: (1) the genetic predispositions factors associated with increased risk of HS in IBD (S*ULT1B1* and *SULT1E1*), (2) the same environmental factor predisposition with increased risk of shooting both HS and CD (i.e., smoking), and, (3) the good response to treatment with anti-TNF-α in HS (as well as in dermatological EIM). Furthermore, HS is an inflammatory pathology of extraintestinal occurrence with increased incidence in patients with IBD in relation to the general population, with similar immune/inflammatory characteristics, fulfilling the criteria for classifying HS as EIM. In this way, we suggest that HS should be considered a dermatological EIM of IBD and treated as such.

Further studies are needed to refine the association between HS and CD, which may have significance in choice of therapies.

## Author contributions

RL responsible for the initial idea developed during the article. MR responsible for assisting in the development of the idea with his extensive experience. PS responsible for collaborating in the production of the article. All authors contributed to the article and approved the submitted version.
